# Acute Abdomen due to Mutual Tangle of Two Small Paratubal Cysts

**DOI:** 10.1155/2018/2351809

**Published:** 2018-10-14

**Authors:** Hiroharu Kobayashi, Shinichi Shibuya, Kentaro Iga, Keiichiro Kato, Airi Kato, Shuhei Terada, Hiroshi Adachi

**Affiliations:** Department of Obstetrics and Gynecology, Seirei Hamamatsu General Hospital, Japan

## Abstract

A 30-year-old woman (gravida 0) visited our hospital with a complaint of right lower abdominal pain. Transvaginal ultrasonography revealed a 5-cm swollen right ovary, which was suspected to be a mature cystic teratoma. Pelvic examination revealed moderate pain. Contrast-enhanced computed tomography showed a 44-mm cystic mass containing fat and calcified material in the right pelvis. Since torsion was suspected, emergent laparoscopic surgery was performed. Intraoperative findings were a swollen right ovary without torsion or congestion. Two small pedunculated 1- and 2-cm diameter paratubal cysts that grew from almost the same place of the ampulla of the right fallopian tube were observed. The thin stalk of the 1-cm paratubal cyst was entangled around the stalk of the 2-cm paratubal cyst, with its head congested. Through a small abdominal laparoscopic incision, the tumor of the right ovary and the two paratubal cysts were excised. Histopathological examination revealed that the right ovarian tumor was a mature cystic teratoma, and the two paratubal cysts had no malignancy. This case showed that only a 2-cm tumor with congestion caused the acute abdomen.

## 1. Introduction

A paratubal cyst is a benign cystic lesion typically located adjacent to a fallopian tube: 76% of cases are paramesonephric (Mullerian), whereas 24% are methothelial [1]. When the adjacent organ is an ovary, the cyst is also called a paraovarian cyst. When the size is large, a paratubal cyst may cause acute abdomen due to torsion to the ovary. Adnexal torsion of a cyst as small as 2 cm has not been reported before. We report a case in which a 1-cm pedunculated paratubal cyst twined around the neighboring 2-cm pedunculated paratubal cyst that was congested, thereby causing acute abdomen.

## 2. Case Presentation

**Figure 1:**
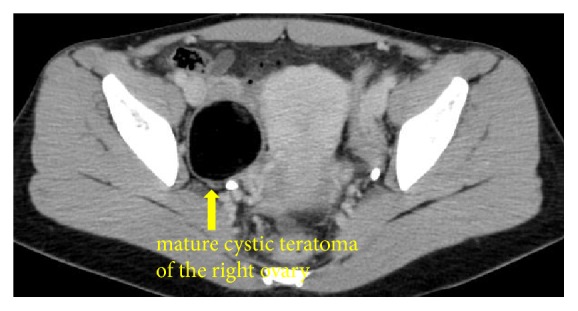
Contrast-enhanced computed tomography image of the pelvic cavity.

**Figure 2:**
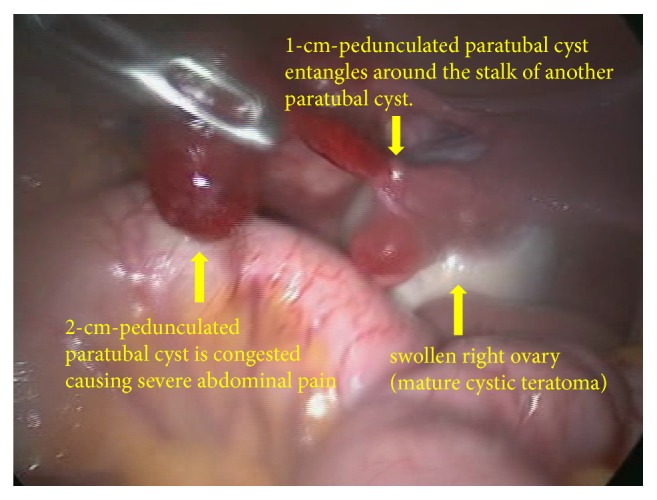
Intraoperative findings from laparoscopy.

**Figure 3:**
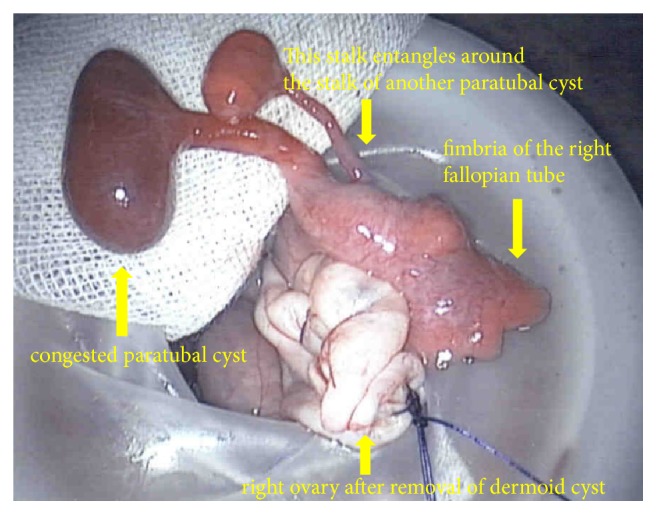
Intraoperative findings from the extracorporeal procedure.

**Figure 4:**
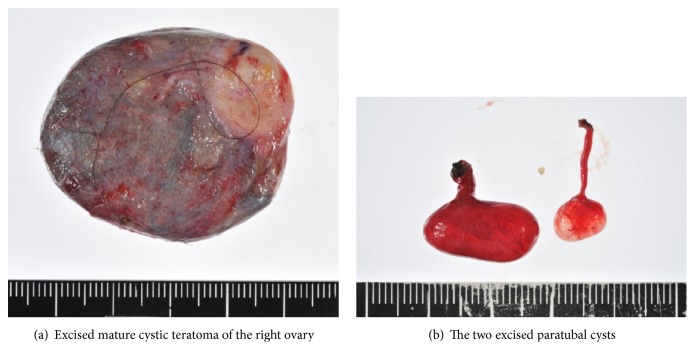


A 30-year-old woman, gravida 0, visited our hospital because of right lower abdominal pain. She had no medical history. She had taken oral contraceptives, and her last withdrawal bleeding started 18 days prior. She reported that the abdominal pain had a gradual onset, and her pain severity alternated between severe and mild. The strongest pain intensity experienced was graded as 8 using the numerical rating scale. Transvaginal ultrasonography revealed a 5-cm swollen right ovary, which was suspected to be a mature cystic teratoma, and the absence of ascites at the Douglas' pouch. Pelvic pain examination demonstrated slightly moderate pain for the condition. Contrast-enhanced computed tomography revealed a 44-mm cystic mass containing fat and calcified material in the right pelvis. There was no finding suggestive of torsion, such as irregular thickening of the cyst wall, whirl sign of the right ovarian vein, and deviation of the affected side of the uterus (Figure 1). However, the possibility of torsion could not be ruled out; thus, an emergent laparoscopic surgery was performed. Intraoperative findings showed a swollen right ovary, but without torsion or congestion. Two small pedunculated 1- and 2-cm diameter paratubal cysts that grew from almost the same place of the ampulla of the right fallopian tube were observed. The thin stalk of the 1-cm paratubal cyst was entangled around the stalk of the 2-cm paratubal cyst, with its head congested, thereby causing the pain (Figures 2 and 3). Through a small abdominal laparoscopic incision, the tumor of the right ovary and the two paratubal cysts were excised (Figure 4). The right ovarian tumor contained fat and hair, and histopathological examination showed that the cyst was a mature cystic teratoma, which was lined with keratinized stratified squamous epithelium and skin appendages, but without an immature component or malignancy. The two paratubal cysts were not malignant.

## 3. Discussion

In a report analyzing 143 cases of adnexal torsion, 84.6% were benign ovarian cysts and paratubal (paraovarian) cysts accounted for 1.4% of cases [2]. Paratubal cysts sometimes twist alone [3–8]. However, in many reports, they were found to twist and involve the adjacent fallopian tube and ovary or the cyst may twine around them [7, 9–18]. The size of the twisted adnexal tumor was reported to be less than 5 cm, 5-10 cm, and more than 10 cm in 32%, 52%, and 16% of cases, respectively [2]. The most common size of the reported twisted paratubal cysts was 5 cm, but some papers reported a 3-cm or follicle-sized twisted paratubal cyst. In this case, a 1-cm pedunculated paratubal cyst coiled around the stem of the adjacent 2-cm paratubal cyst, which caused congestion, thereby resulting in severe abdominal pain that required emergent operation. Torsion may have been caused by the two paratubal cysts, which were adjacent to each other, and the ipsilateral dermoid cyst, which pushed out the fallopian tube, making the release of the torsion difficult.

There has been no report of torsion of a cyst as small as 2 cm in size. This case showed that only a 2-cm adnexal tumor with congestion caused the acute abdomen. It is impossible to preoperatively diagnose torsion in a patient with a small cyst. If the dermoid cyst of the right ovary did not exist, we would not have performed emergent surgery. The pain would have disappeared over time without the need for operation. If the pain was very severe, laparoscopic surgery may have been performed diagnostically. In women with abdominal pain, preoperative identification of the cause of abdominal pain may be difficult through imaging or internal examination; hence, surgery may be necessary.

## References

[1] Samaha M., Woodruff J. D. (1985). Paratubal cysts: Frequency, histogenesis, and associated clinical features. *Obstetrics & Gynecology*.

[2] Erdemoğlu M., Kuyumcuoglu U., Guzel A. I. (2011). Clinical experience of adnexal torsion: Evaluation of 143 cases. *Journal of Experimental Therapeutics and Oncology*.

[3] Yilmaz Y., Ozen I. O., Caliskan D., Dilmen U. (2013). Paraovarian cyst torsion in children: Report of two cases. *Pediatrics International*.

[4] Kiseli M., Caglar G. S., Cengiz S. D., Karadag D., Yilmaz M. B. (2012). Clinical diagnosis and complications of paratubal cysts: Review of the literature and report of uncommon presentations. *Archives of Gynecology and Obstetrics*.

[5] Chauhan S., Blacker C. (2005). Paratubal cyst: a case report.. *West Virginia Medical Journal *.

[6] Dietrich J. E., Heard M. J., Edwards C. (2005). Uteroovarian ligament torsion of the due to a paratubal cyst. *Journal of Pediatric & Adolescent Gynecology*.

[7] Okada T., Yoshida H., Matsunaga T. (2002). Paraovarian cyst with torsion in children. *Journal of Pediatric Surgery*.

[8] Hasuo Y., Higashijima T., Mitamura T. (1991). Torsion of Parovarian Cyst. —Report of Two Cases—. *The Kurume Medical Journal*.

[9] Ottino J., Ricca R. (2016). Paratubal cyst torsion with compromise of the fallopian tube in an adolescent girl. *The American Surgeon*.

[10] Lee J. M., Kim M. J., Kim M. Y. (2014). Gestational weight gain is an important risk factor for excessive fetal growth. *Obstetrics & Gynecology Science*.

[11] Gedam J. K., Rajput D. A., Bhalerao M. V. (2014). Torsion of para-ovarian cyst resulting in secondary torsion of the fallopian tube: A cause of acute abdomen. *Journal of Clinical and Diagnostic Research*.

[12] Kaido Y., Kikuchi A., Kanasugi T., Fukushima A., Sugiyama T. (2013). Acute abdomen due to ovarian congestion: A fallopian tube accompanied by a paratubal cyst, coiling tightly round the ovary. *Journal of Obstetrics and Gynaecology Research*.

[13] Thakore S. S., Chun M. J., Fitzpatrick K. (2012). Recurrent Ovarian Torsion due to Paratubal Cysts in an Adolescent Female. *Journal of Pediatric & Adolescent Gynecology*.

[14] Seshadri S., Morris A., Uchil D., Joloaso A. (2009). Bilateral paratubal cysts with co-existent fallopian tube torsion in an adolescent. *Journal of Obstetrics & Gynaecology*.

[15] Said M. R., Bamigboye V. (2008). Twisted paraovarian cyst in a young girl. *Journal of Obstetrics & Gynaecology*.

[16] Low S.-C. A., Ong C.-L., Lam S.-L., Beh S.-T. (2005). Paratubal cyst complicated by tubo-ovarian torsion: Computed tomography features. *Journal of Medical Imaging and Radiation Oncology*.

[17] Breitowiczi B., Wiebe B. M., Rudnicki M. (2005). Torsion of bilateral paramesonephric cysts in young girls. *Acta Obstetricia et Gynecologica Scandinavica*.

[18] Nelson M. J., Cavalieri R., Graham D., Sanders R. C. (1986). Cysts in pregnancy discovered by sonography. *Journal of Clinical Ultrasound*.

